# Collaborative Efforts in Pediatric Surgery: Lessons from European Randomized Controlled Trials

**DOI:** 10.1055/a-2596-3857

**Published:** 2025-05-21

**Authors:** Anne-Fleur R.L. van Hal, Sara Roman Galdran, Rene M.H. Wijnen, Judith Leyh, Martin Lacher, John Vlot, Omid Madadi-Sanjani

**Affiliations:** 1 Department of Pediatric Surgery, Erasmus MC Sophia Children's Hospital, Rotterdam, South Holland, The Netherlands; 2 Department of Pediatric Surgery, Faculty of Medicine, University of Leipzig, Leipzig, Saxony, Germany; 3 Department of Pediatric Surgery, Hannover Medical School, Hanover, Lower Saxony, Germany; 4 Department of Pediatric Surgery, University Medical Center Hamburg-Eppendorf, Hamburg, Hamburg, Germany

**Keywords:** implementation, international collaboration, multicenter randomized controlled trials, pediatric surgery, rare congenital anomalies

## Abstract

**Background:**

Conducting multicenter randomized controlled trials (RCTs) in pediatric surgery for rare congenital anomalies presents unique challenges, including low patient recruitment, complex regulatory landscapes, and variability in care standards. This paper reflects on the experiences and lessons learned from the MUC-FIRE and STEPS-EA trials, supported by the European Reference Network for Rare Inherited and Congenital Anomalies (ERNICA), to provide guidance for future studies.

**Methods:**

A retrospective review was conducted on the design and execution of these trials, focusing on team composition, endpoint selection, patient recruitment strategies, regulatory compliance, and adaptive methodologies. Insights were derived from study protocols, monitoring reports, and the authors' experiences.

**Results:**

Key factors contributing to trial success included multidisciplinary collaboration, leveraging existing research networks, and defining clear, measurable endpoints. Challenges such as recruitment delays, regulatory hurdles, and variations in care were mitigated through flexible protocols, proactive amendments, and stakeholder engagement. The COVID-19 pandemic amplified these difficulties, necessitating innovative strategies and extended timelines.

**Conclusion:**

The MUC-FIRE and STEPS-EA trials underscore the critical importance of international collaboration, adaptive strategies, and patient-centered approaches in overcoming the complexities of multicenter RCTs. Lessons from these experiences can inform the design and implementation of future trials, ultimately enhancing evidence generation and improving outcomes for children with rare congenital anomalies.

## Introduction

Randomized controlled trials (RCTs) are considered the gold standard for generating high-level evidence in clinical research and are, therefore, generally accepted as the most rigorous way to determine the efficacy of treatments. 1 This is primarily because randomization is a validated strategy to reduce bias, ensuring that study groups are comparable in most characteristics, except for the studied intervention. 2 Although RCTs may not always explain the mechanisms underlying an effect, they allow for systematic analysis of the relationship between interventions and outcomes. While it is accepted that RCTs are the best method to assess efficacy, it is questionable whether they are also the gold standard to evaluate safety. 3 Within RCTs, carefully selected participants undergo interventions in controlled conditions. However, in real life, treatments may reach a wider patient population, including patients with comorbidities or from populations that were unrepresented or excluded from the trial.

Numerous guidelines have been established to standardize RCT designs and strategies, and help clinical scientists overcome certain associated obstacles, such as high costs, time demands, and involvement of representatives of patient groups in the formulation of their hypotheses. Some examples are the Consolidated Standards of Reporting Trials (CONSORT) statement, which provides guidelines for reporting parallel group randomized trials, 4 and the QUOROM statement, which attempts to improve the quality of reports of meta-analyses of RCTs. 5

Although RCTs are widely regarded as the most reliable method for ensuring similar and comparable study groups based on clear and well-established guidelines, they are limited by postrandomization bias, due to non-compliance and dropouts, as well as by reduced generalizability, as a result of complex systems or when conditions are not exactly replicated. 6 Over the past decade, alternative, more pragmatic, trial designs, such as registry-based RCTs, have been explored, 7 8 but are considered to produce a lower level of evidence. Clinical research in pediatric surgery focusing on rare congenital anomalies is confronted with additional challenges, 9 namely low-incidence diseases, small and heterogeneous patient groups, and difficulties obtaining the parents' consent, 10 often in stressful situations, raising questions about ethical justifiability.

To address complex research questions for rare diseases in pediatric cohorts, two international RCTs were established and are currently recruiting patients across Europe, encountering the difficulties associated with rare congenital anomalies. The MUC-FIRE trial aims to evaluate the effects of mucous fistula refeeding on neonates with enterostomies, 11 while the STEPS-EA trial analyzes the safety and effectiveness of intralesional steroid injections to prevent refractory strictures in children after surgical correction of esophageal atresia. 12

As the studies continue, learning from each other's experiences can shape future research by offering valuable insights into the design and execution of international clinical trials in this specialized field. This paper summarizes the authors' experiences in conducting these multicenter RCTs in pediatric surgery, identifying potential pitfalls and proposing best practices.

## Methods

We conducted a retrospective review of the trials' design, focusing on study team formation, hypothesis development, endpoint selection, regulatory compliance, and patient recruitment. Data were collected by exchanging experiences and reviewing trial protocols, ethical approvals, and monitoring reports to identify critical factors impacting the trials' success and challenges.

The RCTs analyzed in this paper, and described below, were endorsed by the European Reference Network for rare Inherited and Congenital Anomalies (ERNICA). ERNICA is one of the 24 European Reference Networks established in 2017 by the European Commission under Directive 2011/24/EU on patients' rights in cross-border health care. 13

**Fig. 1:**
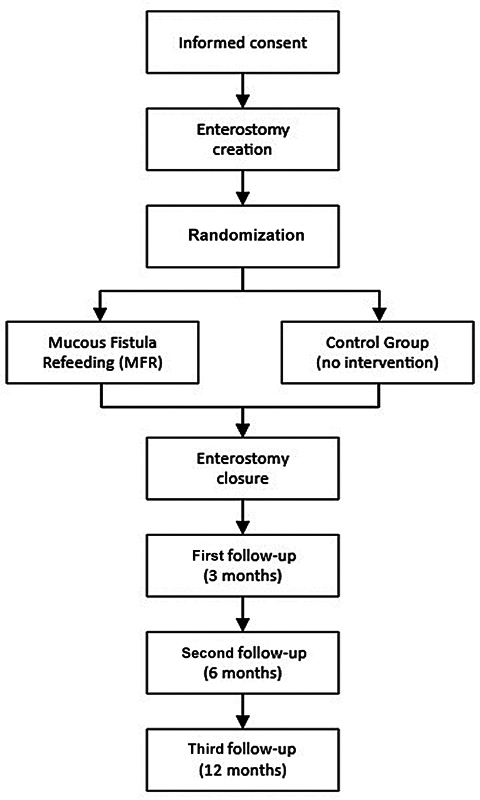
Flowchart of the MUC-FIRE trial study design. 11

**Fig. 2:**
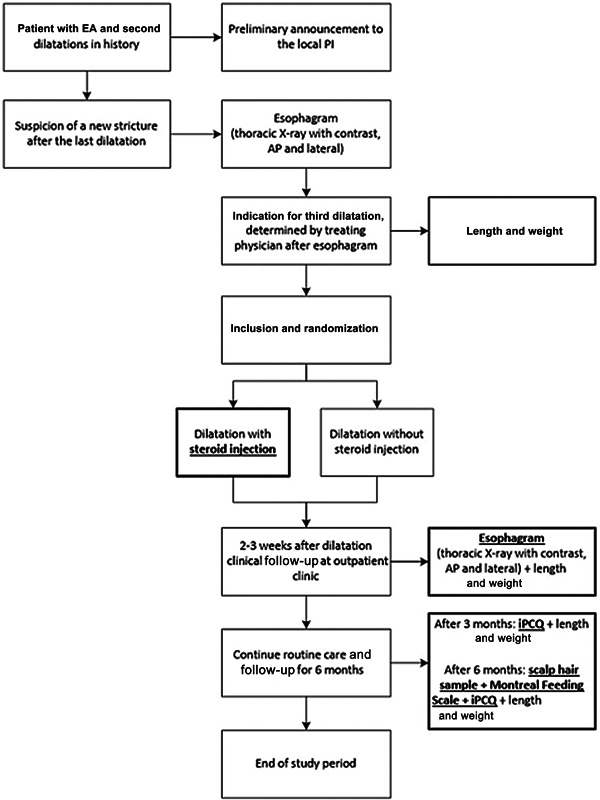
Flowchart of the STEPS-EA trial study design. 12 EA, esophageal atresia; PI, principal investigator; AP, anterior-posterior; iPCQ, iMTA productivity cost questionnaire.

- MUC-FIRE trial
The MUC-FIRE trial (Clinicaltrials.gov registration number: NCT03469609) aims to demonstrate the superiority of mucous fistula refeeding in the management of neonates with enterostomies, mainly including patients after necrotizing enterocolitis and focal intestinal perforation, focusing on the time to full enteral feeds following stoma reversal. 11 A total of 120 infants younger than 366 days of age will be included in the trial, which is currently running in 12 centers across three European countries. The flowchart of the study design is shown in Fig. 1 .
- STEPS-EA trial
The STEPS-EA trial (International Clinical Trials Registry Platform registration number: NTR7726/NL7484) aims to evaluate whether intralesional steroid injections combined with endoscopic dilatation can prevent refractory anastomotic strictures in children with esophageal atresia and thus minimize the number of dilatations needed. 12 A total of 110 children above 3 months of age will be included. The trial is currently running in seven centers across five countries and is still expanding. The flowchart of the study design is shown in Fig. 2 .

In addition, a non-systematic search on Clinicaltrials.gov and the International Clinical Trials Registry Platform was conducted, through which several ongoing European multicenter RCTs in pediatric surgery were identified.

## Results

### Key Considerations for Pretrial Planning and Design

#### Building a Multidisciplinary Research Team

In both the MUC-FIRE and the STEPS-EA trials, research teams are multidisciplinary, which is proving key for the successful design and conduct of the trials. While the core team consists of the Principal Investigator (PI) and clinical research coordinators, statisticians and/or epidemiologists, methodologists, and monitoring and quality boards should be involved early in the conceptualization of the trial. Data managers and data quality monitors are also expected to be mandatory in future RCTs, as on-site monitoring is essential to verify correct information from the participating centers. Existing research facilitation networks, which will be described later in this paper, can assist in connecting the core team with relevant experts. Furthermore, early engagement with clinicians and methodologists from different centers, as well as presenting trials in a preliminary phase at (inter)national congresses or network meetings, can be beneficial when building a multidisciplinary research team.

Last but not least, the value of involving representatives from patient organizations from the start and throughout the trial cycle is now widely recognized. Ensuring that patients' and parents' perspectives are taken into consideration when drafting the protocol can positively impact patient recruitment and retention of trial participants. 14

#### Refining the Hypothesis through Research

Once the clinical issue has been identified, a thorough literature review and search in international clinical trials databases is crucial to define the research gaps, ensure that the study addresses an unmet need, and avoid duplication of existing research. It can shed light on what is already known, highlight any gaps in knowledge, and shape the study's design and methodology, thus ultimately increasing the relevance and impact of the research findings. Literature review should also help develop a hypothesis that mainly analyzes effect-related endpoints.

#### Challenges in Selecting Reliable Primary Endpoints

During the preliminary steps of the MUC-FIRE trial, a literature review revealed that existing retrospective reports primarily focused on weight gain—which can be affected by factors such as tissue edema, time of day, clothing, and the scales used—as a primary endpoint. This shows that endpoints from retrospective studies can introduce unpredictable biases, making them unsuitable for direct application in RCTs. Discussions about the primary endpoint and potential biases took several months and involved methodologists in the debate from the outset.

A primary endpoint must be objectively measurable and consistently repeatable. Objective measurability is important because it has a significant impact on the overall quality of the trial's results. Repeatability is especially crucial in multicenter trials, where various individuals conduct interventions and gather data. Consistency of data across centers and among different colleagues is essential. To accommodate this, the intervention protocols and endpoint analysis methods should be clearly defined. Nevertheless, participating centers should be considered a potential confounding factor. The primary endpoint measurements should also be independent of uncertain factors, such as patient stability during critical care, which could lead to considerable missing data.

#### The Importance of Power Analysis and Managing Dropouts

The assistance of statisticians or epidemiologists early in the trial is necessary to ensure accurate calculations. Power analysis may be based on retrospective analysis of institutional data and experiences—when sufficient numbers are available—or on data from the literature. In addition, it is important to predict the likelihood of dropouts (i.e., study participants who leave the study prematurely). Reasons for dropouts may include loss to follow-up, withdrawal at the request of parents, or excessive missing data. For the MUC-FIRE trial, a dropout rate of approximately 17% was assumed for case number calculation based on statistical analyses. For the STEP-EA trial, the dropout rate was not considered for the power analysis. However, with 15 patients who completed the entire follow-up period, the dropout rate for the STEPS-EA trial at the time of submission of this paper remained 0%.

For both studies, the power calculation was based on a treatment effect with a power of 80% and a two-sided type I error probability of 5% with a log-rank test. 11 12 A probability of committing a Type 1 error means the chances of rejecting the null hypothesis when it is actually true. 15

### Key Considerations for Protocol Development and Implementation in Multicenter Clinical Trials

#### Ensuring Feasibility and Clarity in Multicenter Randomized Controlled Trial Protocols

When drafting a protocol for a multicenter RCT, it is necessary to ensure feasibility across all participating centers. The protocol should be clear enough to maintain scientific value yet include a certain level of flexibility to accommodate differences in the infrastructure of the participating centers.

The study team assembled during the preliminary phase should actively participate in the protocol writing process. To prevent later amendments, researchers from centers planned to participate in the study should also be involved at this point. Moreover, having the protocol reviewed by independent experts who are not involved in the proposed clinical trial can ensure clarity and comprehensibility. This approach minimizes the potential for misinterpretation, even to those not well-versed in the subject matter. Research facilitation networks can provide support with this task.

Table 1 presents the characteristics of several ongoing European multicenter RCTs, obtained from the non-systematic search, providing an overview of the various study designs currently employed.

**Table 1:** Characteristics of European multicenter randomized controlled trials currently recruiting within pediatric surgery

European multicenter RCT	Basic design	Patient population	Intervention	Setting	Study duration
MUC-FIRE trial 11	1:1 randomization between perioperative MFR versus no perioperative MFR	Infants <366 days old with status postileostomy or jejunostomy creation, and all patients with meconium ileus	Perioperative MFR between enterostomy creation and enterostomy closure	Tertiary pediatric surgical centers across Europe	2018–2024
STEPS-EA trial 12	1:1 randomization between intralesional steroid injection prior to balloon dilation versus balloon dilation without any injection	Children ≥3 months in need of third stricture dilatation after EA correction with a distal TEF	Intralesional injection with 10 mg/mL triamcinolone prior to balloon dilation	Tertiary pediatric surgical centers across Europe	2020–2028
RIC-NEC trial 26	1:1 randomization between RIC versus no RIC	Preterm neonates (GA <33 weeks) with NEC diagnosis and current weight ≥750 g	Four cycles of limb ischemia (4-minute via cuff insufflation) followed by reperfusion (4-minute via cuff deflation), repeated on two consecutive days	12 centers in Canada, the United States, Sweden, the Netherlands, the United Kingdom, and Spain	2023–2027
CONNECT trial 27	1:1 randomization between elective surgical resection versus conservative management	Neonates with a prenatally detected CPAM who remain asymptomatic up to inclusion at the age of 3–9 months after confirmation of the index diagnosis on chest CT imaging	Surgical resection of the CPAM lesion after confirmation of diagnosis on chest CT according to the structured report	Pediatric hospitals that routinely care for patients with CPAM and collaborate within the CONNECT consortium	2023–2030
PORTRAIT trial 28	1:1 randomization between PPT versus no PPT	Children born with EA and a distal TEF, and a collapse of the trachea	PPT prior to surgical EA correction	Four expert pediatric surgery hospitals in Europe: GOSH, KI, EMC, and UMCU	2024–2027

Abbreviations: CONNECT, Collaborative Neonatal Network for the first European CPAM Trial; CPAM, congenital pulmonary airway malformation; EA, esophageal atresia; EMC, Erasmus Medical Center, Rotterdam, the Netherlands; GA, gestational age; GOSH, Great Ormond Street Hospital, London, United Kingdom; KI, Karolinska Institutet, Stockholm, Sweden; MFR, mucous fistula refeeding; NEC, necrotizing enterocolitis; PPT, primary posterior tracheopexy; RCT, randomized controlled trial; RIC, remote ischemic conditioning; SickKids, The Hospital for Sick Children, Toronto, Canada; TEF, tracheoesophageal fistula; UMCU, University Medical Center Utrecht, Utrecht, the Netherlands.

#### Navigating Regulatory Compliance in International Clinical Trials

A challenging aspect of conducting international clinical trials is ensuring that participating centers comply with the regulations of the sponsor's institution and the local site. Moreover, adhering to all national and international laws, guidelines, and regulations requires extensive, and often difficult to obtain, documentation. 16 During the preparation phase of a clinical trial, approval is required from several authorities:

- Legal departments
  The sponsor and the participating center must agree on at least a Confidentiality Disclosure Agreement (CDA) and a Clinical Trial Site Agreement (CTA). The CDA safeguards the confidentiality of information exchanged between the sponsor and the clinical trial sites. The CTA outlines the terms governing their collaboration, including responsibilities, financial compensation, and regulatory obligations to be followed by the clinical trial site. Not every country requires specific health insurance for subjects participating in research trials and liability insurance for the medical staff involved in the clinical trial. Therefore, the costs of participating in a clinical trial can vary greatly from country to country.
- Institutional review boards
  Local ethical approval for research involving human subjects is provided by an institutional review board (IRB). In our experience, in some countries, such as Sweden, Denmark, and France, approval from one IRB within the country was sufficient, while other countries, such as Germany, demanded ethical approval from all local participating centers' IRBs. Fortunately, due to legislative changes, from January 1, 2025, multicenter trials in Germany will only be consulted by a single ethics committee.
- Regulatory authorities
The European Union (EU) has a decentralized body, the European Medicines Agency (EMA), whose remit is to protect and promote human and animal health by evaluating and monitoring medicines within the EU and the European Economic Area (EEA). On January 31, 2022, a new Clinical Trials Regulation (CTR) came into application. Under the new Regulation, from January 31, 2023, all applications for interventional clinical trials involving medicinal products for human use in the EU/EEA must be submitted through the Clinical Trials Information System (CTIS). 17 The Regulation enables sponsors to submit a single online application via the CTIS to run a clinical trial in several EU countries, making it more efficient to carry out multinational trials, since EU Member States can evaluate and authorize such applications collectively via one platform.
  In clinical trials involving medicinal products, the investigational agent needs approval and registration in the sponsor's country. If the drug is unavailable on the local market, additional approval must be obtained by the National Competent Authorities before starting the RCT. For the STEPS-EA trial, transition to the CTIS was mandatory despite the fact that the medicinal product in question was approved and widely available.
  Surgical trials that do not study a medicinal product do not fall within the scope of the CTR and must comply with the requirements of national regulatory authorities. This further complicates the regulatory landscape, especially for international trials.

#### Addressing Timeline Challenges and Recruitment Delays

In the past few years, the MUC-FIRE and STEPS-EA trials, as well as many other clinical trials, have faced significant deviations from their originally planned timelines, partly due to the COVID-19 pandemic.

Recruitment of the MUC-FIRE trial was expected to be completed in mid-2023, but only 50% of patients had been recruited by that time. Part of the delay was attributed to the COVID-19 pandemic, which resulted in regulatory restrictions and a decreasing number of (extremely) preterm infants. 18 Additionally, several inconsistencies at the participating centers, primarily related to screening and recruitment, have been identified. These issues could potentially be mitigated by appointing a local researcher with sufficient dedicated time for the trial and providing thorough training during the trial's initiation visit.

The STEPS-EA trial was initially projected to conclude by the end of 2022; however, only 9% of the required patients had been recruited by that time. Also in this case, the COVID-19 pandemic contributed to significant delays, compounded by longer-than-expected approval processes from regulatory authorities. Furthermore, the number of eligible patients was lower than initially anticipated. Resorting to patient registry data can improve the accuracy of estimating the number of eligible trial participants.

Investigators should avoid setting overly optimistic timelines for trial processes, as these can be challenging to achieve in practice. Potential setbacks should be accounted for in the trial timeline, with additional time and funding allocated to accommodate these contingencies.

#### Preparing for Protocol Amendments

Protocol amendments are common in multicenter clinical trials. Therefore, it is essential to actively track potential amendments throughout the study period. Possible amendments include changes in the study team, a prolonged study duration, and modifications to the inclusion or exclusion criteria based on evolving insights, to better align with the study objectives and target population. Additionally, new sites joining the trial may need to draft their own protocol amendment to comply with the requirements of their national authorities.

In 2023, the fourth amendment of the MUC-FIRE study protocol was circulated among all centers and ethics committees, resulting in substantial administrative workload and added costs.

As previously mentioned, the number of eligible patients for the STEPS-EA trial was lower than initially expected. To meet the inclusion criteria, patients must be at least three months old and require a third esophageal dilatation. However, in several cases, the third dilatation occurred before reaching the patient's age of 3 months, meaning the patient was no longer eligible for the study. For this reason, an amendment to the protocol was written in 2024 stating that patients are also eligible when in need of a fourth dilatation and above the age of 3 months. This amendment has increased patient eligibility while maintaining the trial's primary objective of preventing refractory stenosis.

Maintaining flexibility throughout the trials and anticipating potential amendments—and associated costs—from the study's early stages is strongly recommended.

### Strategies for Effective Collaboration and Trial Management in Multicenter Randomized Controlled Trials

#### Taking Advantage of Well-Established Scientific Networks for Collaboration

Establishing collaborations with academic centers that have relevant expertise is crucial. Both the MUC-FIRE and STEPS-EA trials have benefited from endorsement by the ERNICA network, where many centers are familiar with conducting clinical trials. Additionally, presenting the trials at international conferences has been highly effective in fostering collaborations.

Conect4Children (c4c), a large European collaborative network that aims to support the development of new therapies for the pediatric population, provides comprehensive pan-European services for new pediatric research initiatives. 19 Unfortunately, neither the MUC-FIRE nor the STEPS-EA trial could make use of these services, since c4c was still being developed when the trials were set up.

The European Clinical Research Infrastructure Network (ECRIN) may also prove useful when designing and preparing a clinical trial. ECRIN is a non-profit organization that aims to facilitate multinational clinical research. They do so by providing advice and services for the set-up and management of clinical studies in Europe, including legal and regulatory support. 20

#### Reviewing Standard-of-Care Protocols from Potential Collaborators

Standard-of-care protocols may vary between centers, potentially interfering with patient eligibility for the trial. It is recommended that potential differences between participating centers (e.g., standard of care, patient eligibility, structure of the IRB, and legislation) be checked before starting the administrative process of adding them to the trial. This may save a considerable amount of time and costs if it is discovered that patient recruitment might be limited.

#### Variability in Department Roles and Support Needs

Department structures at participating centers can vary significantly. In the Netherlands, for example, most clinical trials are managed by PhD students under the supervision of clinical staff members, while in other countries, the head of the department, consultants, or residents may take on the responsibility for coordinating the clinical trial. In centers with limited research staff, it may be necessary to provide additional support from study coordinators for data assessment and documentation, in accordance with legal regulations. Consequently, it is essential that local researchers are appointed to coordinate the trial at each participating center.

#### Ensuring Accurate Translation and Compliance across Centers

All information texts for patients and their parents should be translated by official translation providers, ensuring that the information remains clear, relevant, and understandable to laypeople. Special expertise is mandatory for the correct translation of medical documents to ensure the quality of information. Depending on the country, other documents required for ethical approval may also need official translation into the local language.

#### Documentation Preparation and Staff Training for First Inclusion

All staff involved in the trial's care pathway must be informed about the RCT and understand the study procedures before the first patient is recruited, preferably during a study site initiation visit. Staff members must also know the inclusion and exclusion criteria to notify the researcher if any eligible patients are identified.

An information kit, containing all relevant documents for conducting the trial, can be provided to participating centers. This documentation should include the study manual, information letter for patients and/or parents, case record forms, and serious adverse event (SAE) forms. This preparation can prevent the researcher or colleague(s) from having to search for documents or information when a patient is included, which can otherwise lead to loss of patients for the trial.

#### Navigating Ethical and Practical Challenges in Informed Consent for Pediatric Clinical Trials

Informed consent in research involving children, and particularly in neonatal clinical trials, raises ethical concerns. 21 22 One of the main reasons is that children cannot voluntarily consent to take part in the clinical trial, and it is therefore the parents' responsibility to make the decision on their behalf. Parents may also feel uncomfortable with this delegated task, which translates into reticence to allow their children to participate in a certain trial.

The Clinical Trials Transformation Initiative (CTTI) conducted research to try to shed light on factors that may influence parents' willingness to enroll their children in clinical trials. 10 Some of the elements directly related to the informed consent procedure included timing and the need for trust, which could be established by involving the child's treating physician, providing empathy training for staff, and using layman's language.

The need to use layman's language is also highlighted in Article 29.2b of the Clinical Trials Regulation, which states that informed consent forms must “be kept comprehensive, concise, clear, relevant, and understandable to a layperson.” 17 However, such adjectives are vague and open to interpretation, and no specific guidelines or metrics are provided to ensure this. To verify that the language, length, and relevance are appropriate, it may be helpful to have the informed consent form for the study reviewed by patient representatives or laypeople. Research facilitation networks and patient organizations can support investigators by connecting them with potential reviewers.

In addition to the above considerations, during the informed consent procedure, researchers should remember to leave enough room for questions from the parents and avoid bias about the research question. Potential risks and burdens need to be thoroughly explained and documented in the electronic patient record.

### Ensuring Safety and Compliance in Clinical Trials: Adverse Events, Monitoring, and Data Safety Oversight

#### Defining and Managing Adverse Events

Adverse events, SAEs, and suspected unexpected serious adverse reactions (SUSARs) must all be clearly defined in the protocol to prevent unnecessary delays in reporting to the regulatory authorities, as well as to reduce excessive bureaucracy in filtering and reporting adverse events. A broad cross-disciplinary coalition of medical societies and patient advocates suggests that including a list of anticipated SAEs that are expected in a trial in the protocol may prevent disproportionate SUSAR reporting requirements, which are known to pose an administrative burden to investigators. 23

During the initiation visit, the entire study team needs to be informed about the (serious) adverse events and the steps to be taken if any occur.

#### The Critical Role of Monitoring in Ensuring Protocol Adherence and Compliance

Monitoring is essential in RCTs to ensure adherence to the protocol, Good Clinical Practice guidelines, and regulatory requirements. Multiple monitoring visits are necessary depending on the trial's risk level (low, moderate, or high). These include at least one initiation visit, one visit after the first inclusion, and one close-out visit.

During monitoring visits, the following are checked: informed consent procedures and forms, Source Data Verification (accuracy and completeness), investigational product accountability, adverse events, protocol compliance, participant records, General Data Protection Regulation (GDPR) adherence, and study team training and support.

External committees or independent researchers can conduct monitoring, but the sponsor can also perform on-site visits.

#### Establishing and Utilizing a Data Safety Monitoring Board

For (drug) interventional studies in humans, a Data Safety Monitoring Board (DSMB) is highly recommended. 24 25 Before starting the clinical trial, several experts in relevant subject areas must confirm their roles within the DSMB. The DSMB will advise on interim safety analyses at predefined times, and its recommendations will determine the study's progress.

### Effective Financial Management and Funding Strategies

Conducting an RCT incurs significant costs, often covered by external funding sources such as individual grants, collaboration subsidies, and European grants. Major expenses include personnel, quality monitoring, ethical submissions, data collection systems, randomization tools, translation services, promotional materials, trial pharmacy and medicinal distribution (if applicable), and publication fees. Extended trial durations may require reapplying for grants and securing additional funding. The MUC-FIRE trial, for example, obtained funding from three grants, while the STEPS-EA trial required an extension with additional costs covered by the sponsor's institution.

In most countries, participating centers must cover the costs of legal affairs and ethics committee review. To reduce the financial burden on these centers and enable them to participate, it is helpful if the sponsor budgets for these costs in advance. Furthermore, the study's efficiency can be significantly increased if a local researcher coordinates the clinical trial on site. Therefore, allocating funds for case payments will benefit the clinical trial.

### Establishing Publication Agreements and Authorship Criteria in Multicenter Trials

Upon trial completion, it is preferable to publish the results as open access to ensure broad dissemination. Clear agreements regarding authorship criteria and the number of authors should be established in advance. For both the MUC-FIRE and STEPS-EA trials, coauthorship for each center is determined by the number of recruited patients.

Establishing publication agreements with participating centers at an early stage helps avoid problems later on. Additionally, incorporating substudies allows centers to propose research ideas, which can act as a motivation to actively recruit patients.

Another option for large multicenter trials is to publish on behalf of the trial collaboration group and list all the authors in detail in an appendix.

## Summary of Challenges and Recommendations

The challenges and recommendations for conducting European multicenter RCTs, drawn from this retrospective review on the design and execution of the MUC-FIRE and STEPS-EA trial, are summarized and presented in Table 2 . This accessible overview can serve as a valuable tool for other investigators working on the design, implementation, and management of RCTs.

**Table 2:** Challenges and recommendations for conducting European multicenter randomized controlled trials

Identified challenges	Recommendations/Best practices
Patient recruitment and retention	Involving representatives from patient organizations from the start and throughout the trial cycle Establishing collaborations with academic centers and making use of existing networks (e.g., European Reference Networks)
Ensuring that the study addresses an unmet need and avoids duplication of existing research	Conducting a thorough literature review Checking international clinical trials databases
Selecting reliable primary endpoints	Selecting primary endpoints that are objectively measurable and consistently repeatable The primary endpoint measurements should also be independent of uncertain factors, such as patient stability during critical care
Inaccurate power analysis	Involving methodologists, statisticians, and/or epidemiologists early in the design of the project
Lack of protocol clarity and feasibility	The protocol should be clear enough to maintain scientific value but include a certain level of flexibility to accommodate differences in the infrastructure of the participating centers. Having the protocol reviewed by independent experts who are not involved in the proposed clinical trial.
Complex regulatory landscape	Drawing on the legal and regulatory expertise of existing research facilitation networks, such as ECRIN
Challenging timelines and recruitment delays	Avoiding overly optimistic timelines Accounting for potential setbacks in the trial timeline, with additional time and funding allocated to accommodate these contingencies
Protocol amendments	Maintaining flexibility throughout the trials and anticipating potential amendments, including cost considerations, from the study's early stages
Variations in standards of care	Checking potential differences between participating centers before starting the administrative process of adding them to the trial
Variability in Department Roles and Support Needs	Appointing local researchers to coordinate the trial at each participating center
Informed consent	Involving the child's treating physician in the informed consent process, providing empathy training for staff, and using layman's language may influence parents' willingness to enroll their children in clinical trials Having the informed consent form for the study reviewed by patient representatives or laypeople. Research facilitation networks can support investigators by connecting them with potential reviewers
Defining and managing adverse events	Defining adverse events, SAEs, and SUSARs in the protocol to prevent unnecessary delays in reporting to the regulatory authorities, as well as to reduce bureaucracy in filtering and reporting adverse events
Authorship issues	Establishing clear agreements regarding authorship criteria and the number of authors in advance

Abbreviations: ECRIN, European Clinical Research Infrastructure Network; SAE, serious adverse event; SUSAR, suspected unexpected serious adverse reaction.

## Limitations

Several limitations of this paper must be addressed. First, most authors are actively involved in the conduct of the MUC-FIRE and/or STEPS-EA trials, which may introduce bias. Second, no specific metrics have been used to evaluate the success of the clinical trials. Future research is needed to develop standardized criteria for measuring the success of randomized clinical trials and to determine whether implementing the recommendations proposed in this manuscript could enhance the measurable success. Finally, this manuscript is based on a retrospective review, which may introduce biases due to the reliance on past data and the subjective interpretation of challenges encountered during the MUC-FIRE and STEPS-EA trials. Nevertheless, this review offers a unique perspective of two major European clinical trials, highlights key lessons learned from their implementation, and gives a structured view with recommendations.

## Conclusion

Despite the complexities and costs, the MUC-FIRE and STEPS-EA trials show the feasibility and importance of international collaboration in conducting RCTs with the potential to produce high-level evidence. To confirm these findings and further improve patient care, additional data, including evidence from RCTs, are needed.

The challenges identified in this paper, along with recommendations to overcome them, are outlined to guide researchers in designing, implementing, and managing future studies more effectively. Sharing experiences from the trials can help build on past learnings to ultimately improve patient care.
